# Analysis of the aging-related biomarker in a nonhuman primate model using multilayer omics

**DOI:** 10.1186/s12864-024-10556-z

**Published:** 2024-06-26

**Authors:** Yunpeng Liu, Shuaiyao Lu, Jing Yang, Yun Yang, Li Jiao, Jingwen Hu, Yanyan Li, Fengmei Yang, Yunli Pang, Yuan Zhao, Yanpan Gao, Wei Liu, Pengcheng Shu, Wei Ge, Zhanlong He, Xiaozhong Peng

**Affiliations:** 1 State Key Laboratory of Respiratory Health and Multimorbidity, National Center of Technology Innovation for Animal Model, National Human Diseases Animal Model Resource Center, NHC Key Laboratory of Comparative Medicine, Beijing Engineering Research Center for Experimental Animal Models of Human Critical Diseases, Institute of Laboratory Animal Sciences, CAMS & PUMC, Beijing, 100021 China; 2 Institute of Medical Biology, Chinese Academy of Medical Sciences, Peking Union Medical College, Kunming, 650031 China; 3 Department of Molecular Biology and Biochemistry, Institute of Basic Medical Sciences, Medical Primate Research Center, Neuroscience Center, CAMS & PUMC, Beijing, 100005 China

**Keywords:** Aging, Biomarkers, LncRNAs, Serum-derived exosomes, Nonhuman primate

## Abstract

**Background:**

Aging is a prominent risk factor for diverse diseases; therefore, an in-depth understanding of its physiological mechanisms is required. Nonhuman primates, which share the closest genetic relationship with humans, serve as an ideal model for exploring the complex aging process. However, the potential of the nonhuman primate animal model in the screening of human aging markers is still not fully exploited. Multiomics analysis of nonhuman primate peripheral blood offers a promising approach to evaluate new therapies and biomarkers. This study explores aging-related biomarker through multilayer omics, including transcriptomics (mRNA, lncRNA, and circRNA) and proteomics (serum and serum-derived exosomes) in rhesus monkeys (*Macaca mulatta*).

**Results:**

Our findings reveal that, unlike mRNAs and circRNAs, highly expressed lncRNAs are abundant during the key aging period and are associated with cancer pathways. Comparative analysis highlighted exosomal proteins contain more types of proteins than serum proteins, indicating that serum-derived exosomes primarily regulate aging through metabolic pathways. Finally, eight candidate aging biomarkers were identified, which may serve as blood-based indicators for detecting age-related brain changes.

**Conclusions:**

Our results provide a comprehensive understanding of nonhuman primate blood transcriptomes and proteomes, offering novel insights into the aging mechanisms for preventing or treating age-related diseases.

**Supplementary Information:**

The online version contains supplementary material available at 10.1186/s12864-024-10556-z.

## Background

Aging, characterized by time-dependent functional decline at cellular and organismal levels, is a primary risk factor for prevalent diseases, including cancer [1], cardiovascular disease [2], and neurodegeneration [3]. High-throughput sequencing of human samples [4] and various animal models [5, 6] has revealed numerous age-related genes mechanistically linked to longevity. Findings from the past decade have emphasized the translatability of the molecular mechanism of aging to primates [7, 8], with implications for human aging biology [9]. Rhesus monkeys, sharing 93.5% genomic identity with humans [10], along with analogous physiological and behavioral features [9, 11], serve as a pertinent model for human aging, exhibiting an increased incidence of age-related pathological conditions. Therefore, understanding aging mechanisms in nonhuman primates (NHPs) may provide additional targets for preventing or treating age-related diseases.

The complex mechanism of aging encompasses genomic instability, telomere attrition, epigenetic alterations, proteostasis loss, disabled macroautophagy, deregulated nutrient sensing, mitochondrial dysfunction, cellular senescence, stem cell exhaustion, altered intercellular communication, chronic inflammation, and dysbiosis [12]. Major advances in high-throughput sequencing technology [13, 14] and mass spectrometry (MS)-based proteomics [15, 16] have enabled the identification of products at various expression levels with increased accuracy and reproducibility. Thus, numerous studies have applied genomic, transcriptomic (including mRNA, miRNA, circRNA, and lncRNA), and proteomic assays (collectively termed “multiomics”) in aging research [17, 18]. For example, computational integration of the aging proteome with single-cell transcriptomes has enabled the construction of an unbiased reference map of the aging lung [19]. Furthermore, the regulatory mechanisms of circRNAs [20, 21] and lncRNAs [22, 23] have been reported in aging mammalian tissues.

Unlike specific tissues, blood contains RNAs and proteins from nearly all cell types and tissues. Multiple studies have demonstrated the potential rejuvenation of various tissues, such as muscle, liver, heart, pancreas, kidney, bone, and brain tissues, through the interconnection of circulatory systems in old mice with those of their younger counterparts [24]. Identifying varying expression levels of key regulators in blood can contribute to understanding organismal aging mechanisms [25]. Nevertheless, comprehensive exploration of these mechanisms, particularly in NHPs, remains limited, and more information is needed.

**Fig. 1:**
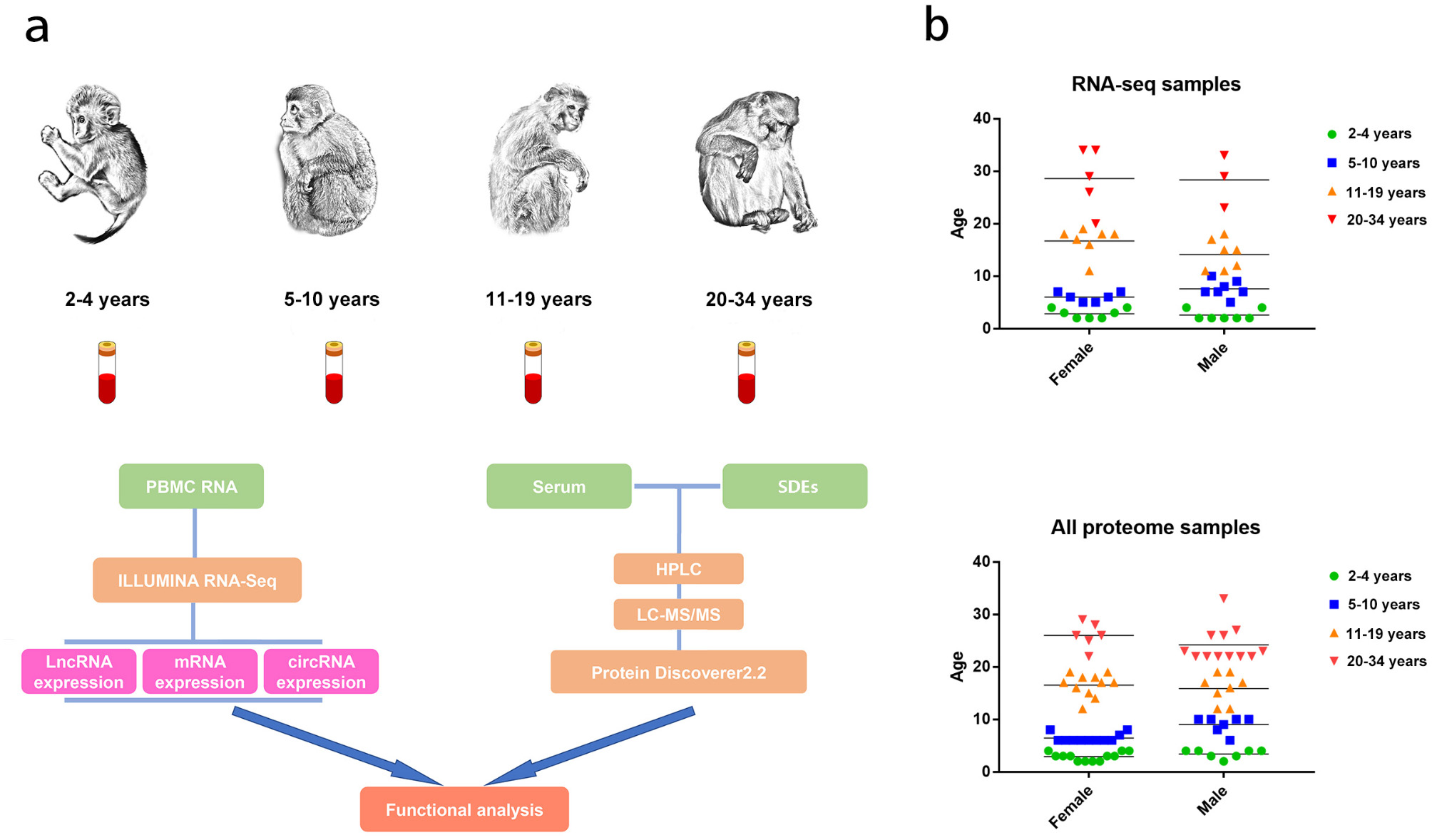
Comprehensive catalog of RNA genes and proteins in rhesus monkey blood. **a**. Illustration of the experimental design and bioinformatics analysis pipeline. **b**. Ages and numbers of monkeys used in the transcriptomics and proteomics analyses. The horizontal line represents the average age of different groups of rhesus monkeys

In this study, we sequenced the blood transcriptomes (mRNA, lncRNA, and circRNA) and proteomes [serum and serum-derived exosomes (SDEs)] in four age groups of rhesus monkeys to examine transcription and protein level changes (Fig. 1a). Through multiomics analysis of blood, we provide novel insights into the molecular foundations of aging. Additionally, we identify eight candidate aging biomarkers with applicability as blood-based biomarkers for detecting brain aging.

## Methods

### Animals and sample collection

Whole blood samples were collected from 282 rhesus macaques provided by the Medical Primate Research Center of Institute of Medical Biology, Chinese Academy of Medical Sciences. Information on the growth, development, and reproduction of rhesus monkeys in captivity was provided by the Medical Primate Research Center and obtained from published literature [26]. The monkeys used in the study were groups into four categories: 2–4 years, 5–10 years, 11–19 years, and 20–34 years. Sample collection were performed under anesthesia. All animals were anaesthetized with Zoletil^®^50 (4 mg/kg, Virbac) and administrated intramuscularly. We randomly selected 49 out of 282 samples for RNA extraction as well as 74 samples for serum and SDE protein extraction. Brain samples were obtained from the Primate Laboratory Animal Biobank, Medical Primate Research Center of Institute of Medical Biology, Chinese Academy of Medical Sciences.

### RNA-seq library construction and sequencing

Whole blood, preserved in PAXgene Blood RNA Tubes (PreAnalytiX, Qiagen, Hombrechtikon, Switzerland), was stored at − 80 °C. Total RNA extraction was performed using the PAXgene Blood miRNA Kit (PreAnalytiX, Qiagen) following the manufacturer’s instructions. RNA purity, concentration, and integrity were determined using a NanoPhotometer spectrophotometer (IMPLEN, Westlake Village, CA), a Qubit 2.0 Fluorometer with the Qubit RNA Assay Kit (Life Technologies, Carlsbad, CA), and the Bioanalyzer 2100 System with the RNA Nano 6000 Assay Kit (Agilent Technologies, Santa Clara, CA), respectively.

For RNA sample preparations, 5 µg of RNA per sample served as input material. Ribosomal RNA was initially removed using the Epicentre Ribozero^®^ rRNA Removal Kit (Epicentre, USA), followed by ethanol precipitation for cleaning rRNA-free residues. Subsequently, linear RNA underwent digestion with 3 U of RNase R (Epicentre) per microgram of RNA. The NEBNext^®^ Ultra™ Directional RNA Library Prep Kit for Illumina^®^ (NEB, USA) was employed for generating sequencing libraries following the manufacturer’s recommendations. Briefly, fragmentation was performed using divalent cations under an elevated temperature in NEBNext First Strand Synthesis Reaction Buffer (5X). First-strand cDNA was synthesized using the random hexamer primer and M-MuLV Reverse Transcriptase (RNaseH-). Subsequently, second-strand cDNA was synthesized using DNA Polymerase I and RNase H. In the reaction buffer, dTTP was replaced by dUTP. Remaining overhangs were converted into blunt ends via exonuclease/polymerase activities. After adenylation of DNA fragments’ 3′ ends, an NEBNext Adaptor with a hairpin loop structure was ligated in preparation for hybridization. To preferentially select 150–200 bp cDNA fragments, library fragments were purified using an AMPure XP system (Beckman Coulter, Beverly, USA), and 3 µL of USER Enzyme (NEB, USA) was incubated with size-selected, adaptor-ligated cDNA at 37 °C for 15 min, followed by 5 min at 95 °C prior to polymerase chain reaction (PCR). PCR was then performed using Phusion High-Fidelity DNA Polymerase, Universal PCR primers, and Index (X) primers. Finally, the products were purified using the AMPure XP system, and library quality was assessed using the Agilent Bioanalyzer 2100 system.

Index-coded samples were clustered using the cBot Cluster Generation System with the TruSeq SR Cluster Kit v3-cBot-HS (Illumina) according to the manufacturer’s instructions. After cluster generation, the lncRNA, mRNA, and circRNA library preparations were sequenced on an Illumina HiSeq 4000 platform, generating 150 bp paired-end reads.

### RNA-seq raw data filtering, mapping, and alignment statistics

Clean reads were obtained after removing adaptor-containing reads, poly-N–containing reads, and low-quality reads from the raw data. Clean reads were aligned to the Ensemble genome (Macaque 8.0.1) using Bowtie (version 2.2.8) [27] with its default parameters. The transcriptome of each sample was constructed using Cuffdiff (version 2.1.1) [28]. Fragments per kilobase of exon per million reads mapped (FPKM) was used to quantify the expression level of mRNAs and lncRNAs, whereas transcripts per million (TPM) was used to determine the circRNA expression level. Differences between groups were determined using the DESeq2 R package.

### LncRNA and circRNA prediction

LncRNAs were identified following six steps:

1. Paired-end clean reads were aligned to the macaque genome using HISAT2 (version 2.0.4) with “-rna-strandness RF,” and the mapped reads of each sample were assembled using StringTie (version 1.3.1) [29], taking a reference-based approach.
2. Cuffcompare, embedded in Cufflinks, was used to combine all assembled transcripts.
3. Transcripts of < 200 bp in length or those with < 2 exons were removed.
4. Transcripts exhibiting FPKM < 0.5 were also removed.
5. Cuffcompare was used to compare newly identified lncRNA transcripts with known macaque lncRNA transcripts, and novel transcripts in intergenic and antisense regions were reserved as candidate lncRNAs.
6. The coding potential of transcripts was assessed using four software programs: CNCI, CPC, Pfam, and phyloCSF [30–33], and transcripts lacking coding potential were classified as novel lncRNAs. Both novel lncRNAs and known lncRNAs were selected for the final analysis.

CircRNAs were detected and identified using find_circ (version 1.1) [34] and CIRI2 (version 1.2) [35].

### DE mRNA, lncRNA, and circRNA analyses

DE mRNAs and lncRNAs were identified using Cuffdiff (version 2.1.1) [28], with DE transcripts defined as those with a q-value of < 0.05. DE circRNAs were analyzed using the DESeq2 R package (version 1.8.3). *P* < 0.05 was set as the threshold for significance. Different groups were compared to identify DE mRNAs, lncRNAs, and circRNAs, which were subsequently combined into one DE union set. Short Time-series Expression Miner (STEM) software was used to cluster DE RNAs according to their temporal expression profiles, and those profiles achieving *p* ≤ 0.05 were considered significantly enriched.

### RT-qPCR and PCR

Total RNA was reverse-transcribed into first-strand cDNA using the High-Capacity cDNA Reverse Transcription Kit (Applied Biosystems). RT-qPCR was performed using SsoFast™ EvaGreen^®^ Supermix (BIO-RAD), with validation conducted using three biological replicates. The primer pairs used are presented in Additional file 18. Relative quantities of immunoprecipitated DNA fragments were calculated, referencing a standard curve generated using input DNA serial dilutions. Data were acquired from three independent amplifications. For circRNA junction sequence confirmation, PCR was performed using Q5 Hot Start High-Fidelity 2X Master Mix (NEB) with the primer pairs presented in Additional file 19. PCR products underwent gel purification and were submitted for Sanger sequencing.

### Isolation of serum and SDEs

Serum samples were pooled and divided into four groups. Exosomes were isolated through ultracentrifugation (UC) with total exosome isolation reagent. Cell debris was removed from serum using UC at 2,000 *g* and 4 °C for 30 min. The supernatant was centrifuged at 12,000 *g* and 4 °C for 40 min, filtered through a 0.22 μm membrane filter, and diluted using an equal volume of phosphate-buffered saline (PBS). Diluted serum was then transferred into ultracentrifuge tubes, and UC (Beckman Optima L-100XP) was performed at 110,000 *g* and 4 °C for 120 min. The pellet was gently washed once with PBS without disturbance, after which it was dissolved in 50 µL of 8 M urea. Total protein concentration was determined using a NanoDrop 2000 spectrophotometer (Thermo Scientific). Lysates from each group were diluted to 1 mg/mL using 8 M urea for tandem mass tag (TMT) labeling. For exosome isolation with total exosome isolation reagent, the required volume of pooled serum was diluted with an equal volume of PBS to decrease viscosity, and 0.2 volumes of total exosome isolation reagent were added. The serum/reagent solution was vortexed until homogenized, followed by incubation at 4 °C for 30 min. Subsequently, samples were centrifuged at 10,000 *g* for 10 min at room temperature, and the supernatants were discarded. The pellet from every 100 µL serum sample was resuspended in 25 µL of PBS for western blot analysis. A fraction of the resuspended exosomes was lysed with radioimmunoprecipitation assay (RIPA) buffer, and protein concentration was determined using a BCA Protein Assay Kit.

### Depletion of high-abundance proteins from serum

A Seppro IgY14 LC-2 column (Sigma, USA) was used to remove high-abundance proteins (HAPs), including HSA, IgG, fibrinogen, transferrin, IgA, IgM, haptoglobin, alpha2-macroglobulin, alpha1-acid glycoprotein, alpha1-antitrypsin, Apo A-I HDL, Apo A-II HDL, complement C3, and LDL (ApoB), from serum. Serum samples were diluted with Tris-buffered saline (TBS; 10 mM Tris-HCl, 150 mM NaCl, pH 7.4) before injection (120 µL) into the column. HAP depletion and column reactivation were accomplished using the following chromatography method: setting an initial flow rate of 0.2 mL/min for 17 min using TBS as a dilution buffer (10 mM Tris-HCl, 150 mM NaCl, pH 7.4), washing the column at a flow rate of 1.5 mL/min for 5 min using stripping buffer (0.1 M glycine-HCl, pH 2.5), neutralizing the column at a flow rate of 1.5 mL/min for 14 min using neutralization buffer (0.1 M Tris-HCl, pH 8.0), and balancing the column at a flow rate of 1.5 mL/min for 6 min using dilution buffer. HAPs were held on the column, whereas proteins in the flow-through fraction were collected and concentrated using a 3 kD ultrafiltration tube (Millipore, USA). Concentrated serum flow-through fractions were diluted with an equal volume of 8 M urea lysis buffer, and 50 µg of protein from each group was used for subsequent MS detection.

### MS measurement and label-free analysis

TMT-labeled proteins underwent fractionation into 12 fractions using an Xbridge BEH300 C18 column (Waters, MA, USA) on a Thermo UltiMate 3000 UPLC workstation (Thermo Fisher Scientific, MA, USA). Each fraction was dried and reconstituted in 0.1% trifluoroacetic acid before MS analysis. Liquid chromatography with tandem mass spectrometry (LC-MS/MS) was performed on a Thermo Orbitrap Fusion Lumos mass spectrometer (Thermo Scientific) in positive-ion mode. Raw MS/MS data were collected and analyzed using Xcalibur 3.0 software (Thermo Fisher Scientific). Protein identification and quantification were performed using Proteome Discoverer 2.2 software (Thermo Scientific). The *Macaca mulatta* FASTA database (released on November 12, 2018) was used for MS/MS spectrum matching, setting the credible threshold as exp. q-value: combined < 0.01 for high confidence.

### Western blot analysis

Approximately 20 µg of protein underwent sodium dodecyl sulfate–polyacrylamide gel electrophoresis and electrotransfer to a polyvinylidene difluoride membrane (Millipore). Membranes were blocked with 5% nonfat milk in TBS-T (TBS plus 0.5% Tween) for 1 h, followed by incubation with monoclonal antibodies against A2M (1:1000; Abcam), SERPINA3 (1:1000; Abcam), and transferrin (1:10,000; Abcam). Subsequent incubation involved an horseradish peroxidase–conjugated rabbit anti-goat antibody (1:5000; ZSGB-BIO). ImageJ was used to quantify western blots.

### KEGG enrichment analysis

KOBAS software [36] was used to identify enriched Kyoto Encyclopedia of Genes and Genomes (KEGG) pathways in serum DEPs, SDE DEPs, DE mRNAs, DE lncRNAs, and DE circRNAs [37]. A hypergeometric p-value of < 0.05 was considered significant.

### GO enrichment analysis

GO functional enrichment analysis and dataset comparisons were performed using FunRich [38]. GO analysis of annotated proteins was conducted to determine the involved cellular components. Enriched terms were ranked based on p-values (hypergeometric test) using FunRich. GraphPad Prism software (La Jolla, CA) was employed to plot graphs when comparing datasets. For comparisons of datasets within a single GO term, significant protein distribution differences were assessed using the chi-square test via GraphPad Prism. Datasets with a p-value of < 0.05 were considered significant.

## Results

### Transcriptome atlas of rhesus monkey whole blood

Rhesus monkeys, aged 2–34 years (Fig. 1b), were categorized into four age groups: 2–4 years, 5–10 years, 11–19 years, and 20–34 years. Blood biochemical indexes and routine tests confirmed the good physical condition of the examined rhesus monkeys (Additional file 9). For an in-depth assessment of transcriptome variations, RNA-seq was used to profile mRNA, lncRNA, and circRNA transcriptomes from 49 whole blood samples across the four groups. Using the Illumina sequencing platform, we generated 74.8 million high-quality pair-end reads, averaging 18.7 million reads per group. Approximately 94.9% of these reads aligned to the rhesus monkey genome Ensemble Macaque 8.0.1 (Additional file 10).

In total, 31,620 mRNAs from 13,936 genes were detected, identified, and quantified (Additional file 11). Regarding lncRNA expression patterns, ab initio transcript assembly and sequential filtering steps (refer to the “Methods” section), were used to identify 13,274 lncRNAs (Additional file 12), including 996 (9.7%) originating from antisense regions (Additional file 1). Dynamic changes in circRNA expression were characterized based on theoretical predictions, with 3,616 circRNAs detected using find_circ [34] and CIRI2 [35] (Additional file 13). Predominately, circRNAs were exonic (85.3%), with only a small proportion containing introns and unannotated intergenic regions (Additional file 1). Randomly selected circRNAs were subjected to PCR and Sanger sequencing, with the sequencing results being highly consistent with RNA-seq findings (Additional file 2).

### LncRNAs are highly expressed in the key aging period

**Fig. 2:**
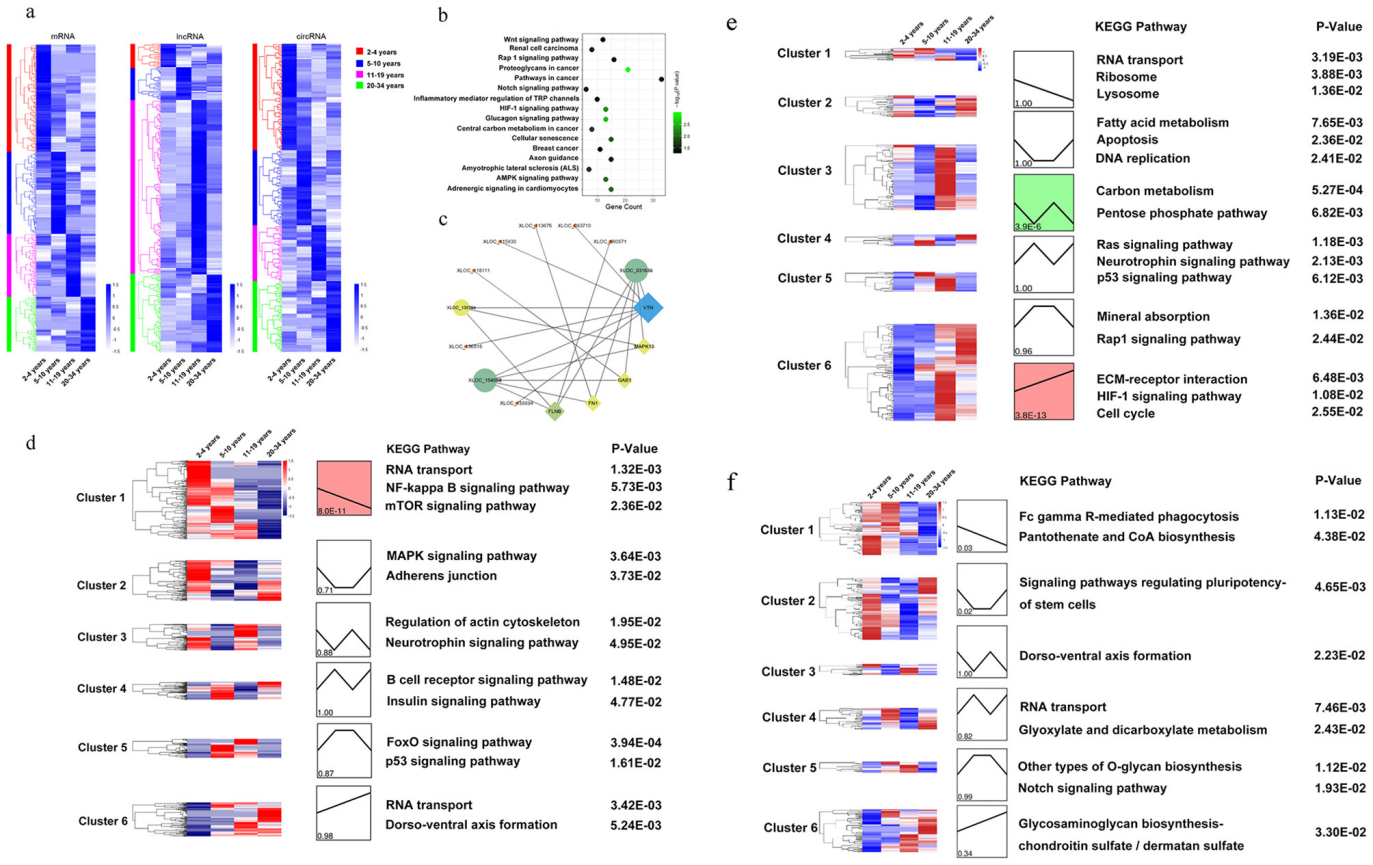
Expression patterns of mRNAs, lncRNAs, and circRNAs. **a**. Hierarchical clustering heat map of all DE mRNAs, lncRNAs, and circRNAs across the four experimental groups. **b**. Enriched categories for highly expressed lncRNAs in 11–19 years. **c**. Coexpression network of lncRNAs and mRNAs associated with the proteoglycans in cancer KEGG pathway. Circles represent lncRNAs, rhombuses represent mRNAs, and shape size represents statistical significance. **d-f**. DE mRNAs, DE lncRNA and circRNA were clustered into six groups using STEM, with the colors in each cluster indicating statistical significance (*p* ≤ 0.05; red, upregulated; blue, downregulated). Expression values, representative KEGG pathways, and corresponding enrichment p-values are shown

To investigate the expression patterns of mRNAs, lncRNAs, and circRNAs, pairwise comparisons were conducted among the four age groups. These revealed 532 differentially expressed (DE) mRNAs, 250 DE lncRNAs, and 233 DE circRNAs between any two stages. As shown in the heatmap in Fig. 2a, unlike mRNAs and circRNAs, an abundance of highly expressed lncRNAs was observed (136; 54.4%) in 11-19 years, which represents a critical aging period. This suggests that lncRNAs play an important role in aging.

A correlation matrix was generated between 13,274 lncRNAs and 31,620 mRNAs by computing Pearson correlation coefficients for all pairwise combinations based on their expression in our transcriptomes. In total, 3,652 coexpression pairs were detected between 136 lncRNAs and 1,103 mRNAs. KEGG pathway analysis for the 136 lncRNAs interacting with mRNAs (*p* < 0.05) revealed their extensive involvement in renal cell carcinoma, proteoglycans in cancer, cancer pathways, HIF-1 signaling, central carbon metabolism in cancer, cellular senescence, breast cancer, axon guidance, and amyotrophic lateral sclerosis (Fig. 2b). Regarding proteoglycans in cancer (*p* = 1.08e-03), the vitronectin gene interacted with seven lncRNAs, and *XLOC_031636* interacted with five mRNAs (Fig. 2c).

### Dynamic transcriptome changes during aging in rhesus monkeys

Cluster analysis with STEM was used to categorize all DE mRNAs, lncRNAs, and circRNAs into six distinct groups [35], revealing linear patterns (clusters 1 and 6) as well as several nonlinear trajectories (clusters 2–5). KEGG pathway analysis for each cluster identified representative KEGG terms (*p* < 0.05), highlighting distinct yet coordinated changes in biological processes during aging (Additional file 14 and 15). For DE mRNAs, cluster 1 was prominent (168; 31.6%), with these mRNAs primarily enriched in pathways including RNA transport, NF-kappa B signaling, and mTOR signaling (Fig. 2d). Clusters 4 and 5 were enriched in B cell receptor signaling, insulin signaling, FoxO signaling, and p53 signaling. Among DE lncRNAs, clusters 3 and 6 were prominent (160; 64%), with these lncRNAs being mainly enriched in carbon metabolism, the pentose phosphate pathway, extracellular matrix–receptor interaction, the cell cycle, and HIF-1 signaling (Fig. 2e). Regarding DE circRNAs, in clusters 1 and 2, which accounted for over half of all DE circRNAs (118; 50.6%), pathways associated with Fc gamma R-mediated phagocytosis, pantothenate and CoA biosynthesis, transcriptional misregulation in cancer, and signaling regulating stem cell pluripotency were enriched (Fig. 2f). These results emphasize the distinct yet orchestrated transcriptome changes that occur during aging.

### Exosomal proteins as the main components of serum proteins

For a comprehensive understanding of protein level changes, parallel to RNA-seq, TMT-based quantitative MS was employed on serum and SDEs. Electron microscopy images confirmed the intact morphology of SDEs used in our study (Additional file 3) Raw spectral data were interpreted using Proteome Discoverer 2.2, with 1,270 and 2,162 proteins found to have at least one unique peptide in serum and SDEs, respectively, given a false discovery rate (FDR) of < 0.01 (Additional file 16 and 17).

**Fig. 3:**
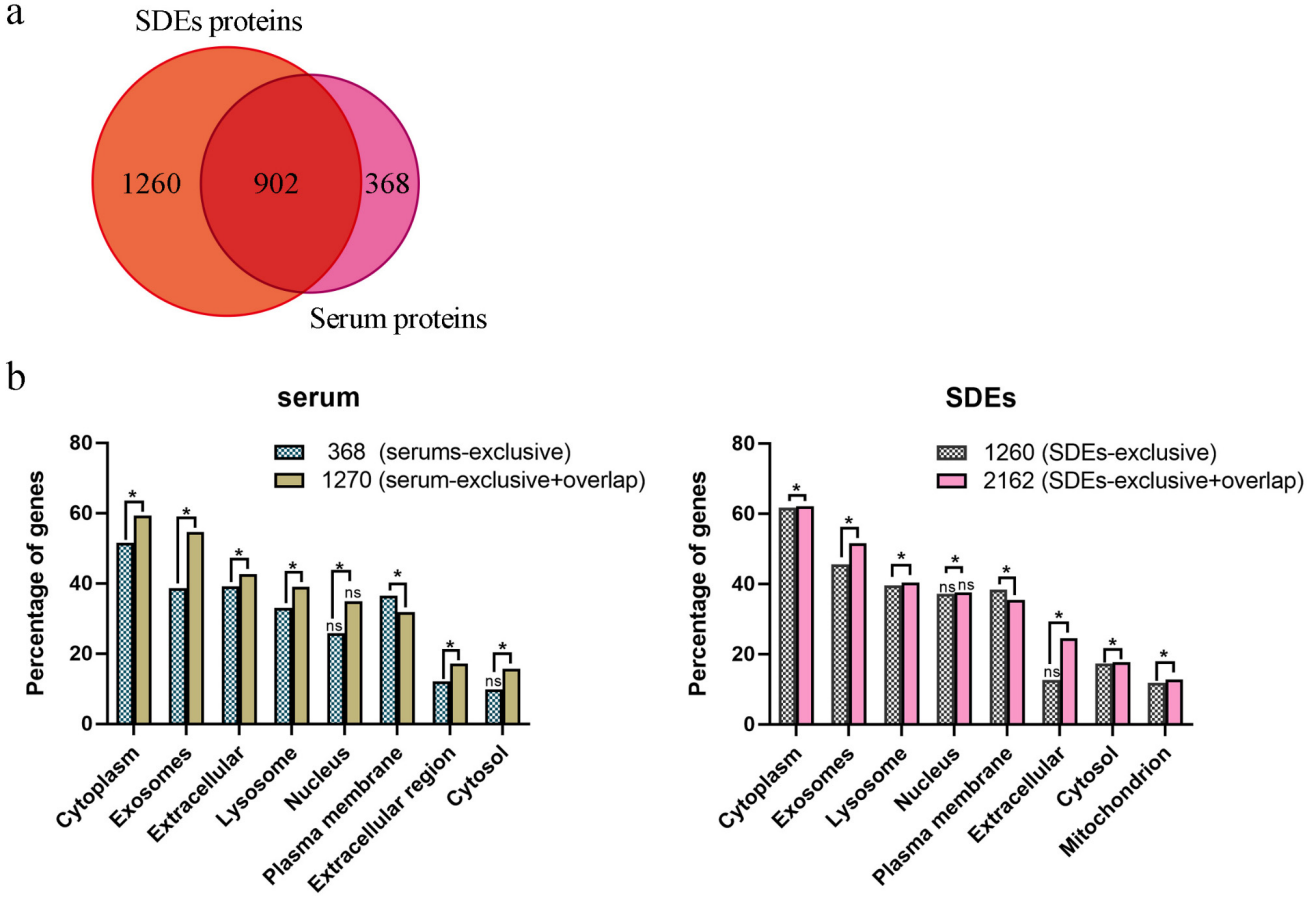
Comparison of serum and SDE proteomes. **a**. Venn diagram comparing uniquely identified and shared proteins between serum and SDEs. **b**. Functional annotations of cellular components for the identified proteins, comparing all 1,270 proteins identified in serum with 368 proteins identified exclusively in SDEs. Additionally, all 2,162 proteins identified in SDEs were compared with 1,260 proteins identified exclusively in the sample. Enriched terms were ranked according to p-values (hypergeometric test) for both datasets. GO terms not significantly enriched are indicated by “ns.” Within individual GO terms, the distribution of annotated proteins was compared using the chi-square test. Datasets exhibiting a significant difference are indicated by “*.” *P* < 0.05 was considered significant for both the hypergeometric test and chi-square test

The total number of proteins identified was higher in SDEs than in serum, with an overlap of 902 gene symbols between the two (Fig. 3a). Functional enrichment analysis of cellular components using FunRich 3.1.3 [38] revealed a high percentage of proteins in both serum and SDEs associated with various Gene Ontology (GO) terms, such as “cytoplasm,” “exosomes,” and “extracellular” (Fig. 3b). To assess the impact of overlapping proteins identified in the enrichment analysis, we compared nonoverlapping proteins (368 and 1,260 in serum and SDEs, respectively) with the proteins identified in the entire sample (1,270 and 2,162 in serum and SDEs, respectively). Excluding overlapping proteins from the GO analysis significantly reduced the number of serum and SDE proteins associated with the term “exosomes,” indicating that overlapping proteins, i.e., most proteins in the serum, are likely derived from exosomes, whereas 368 proteins unique to the serum are likely unassociated with exosomes. This indicates that SDEs are a more suitable option than serum for blood proteome studies.

### Dynamic SDE protein changes during aging in rhesus monkeys

**Fig. 4:**
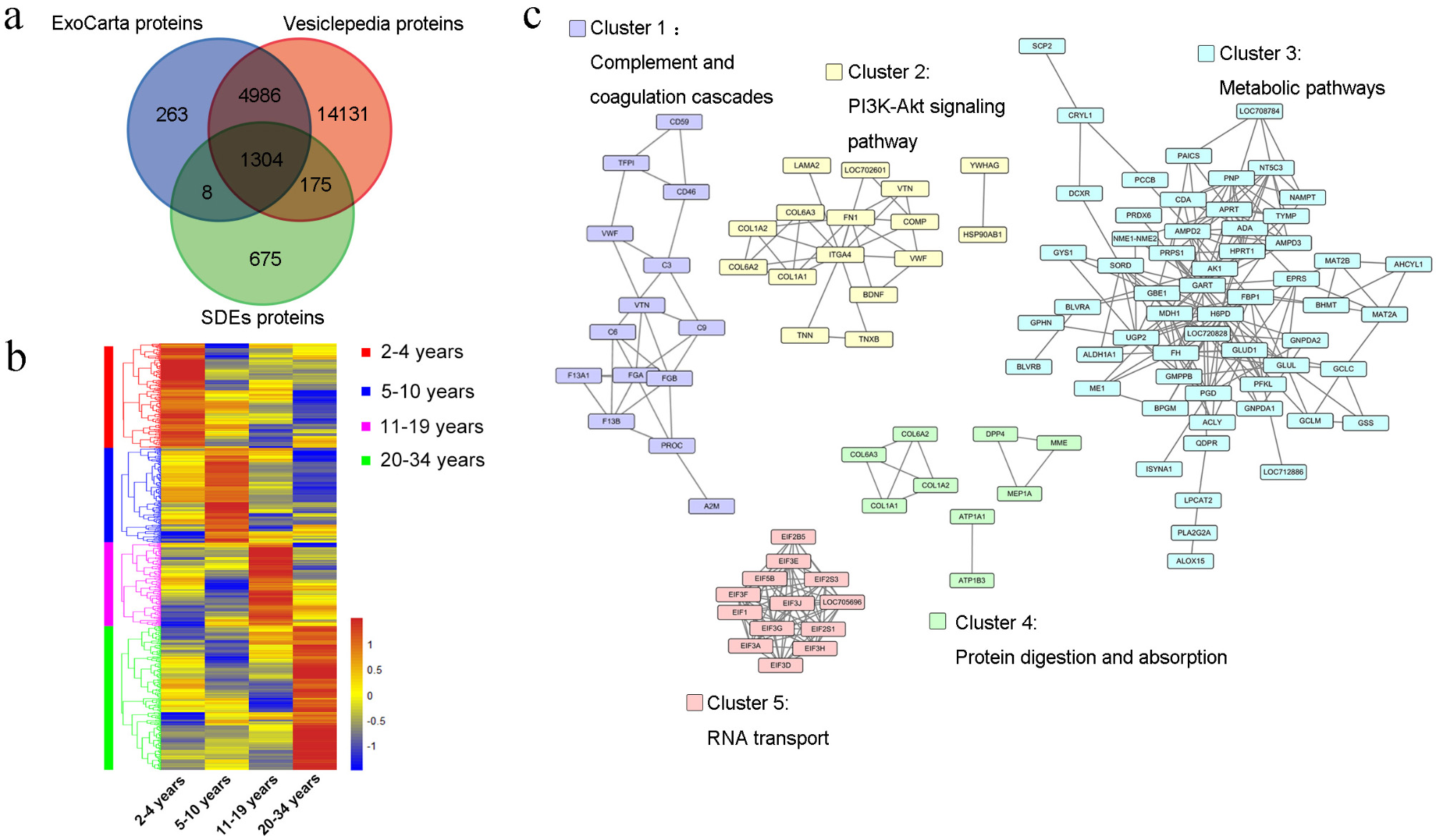
Proteome landscape of SDEs in rhesus monkeys. **a**. Venn diagram showing the overlap between the SDEs identified in the present study and those from the ExoCarta and Vesiclepedia databases. **b**. Hierarchical clustering heat map of all DEPs in SDEs across four age groups. **c**. Four clusters of DEPs in SDEs were enriched in KEGG pathways

Given the crucial role of exosome proteins in systemic aging [39], we investigated dynamic changes in SDE proteins during aging. Comparisons of the 2,162 SDE proteins with those in the Vesiclepedia database [40] and ExoCarta database [41] identified 1,487 reported proteins, including 675 novel proteins (Fig. 4a).

In total, 654 differentially expressed proteins (DEPs; cutoff ratio ≥ 1.50 or ≤ 0.67) between any two stages were identified in SDEs (Fig. 4b). Using the web-based STRING tool (http://string-db.org) to create comprehensive DEP networks with an FDR < 0.05 based on the KEGG pathway analysis and Cytoscape [42] for network visualization, we visualized protein–protein interactions. Five major clusters emerged in the SDEs (Fig. 4c). Cluster 1 was enriched in complement and coagulation cascades related to immunity; cluster 2 was enriched in the PI3K-Akt signaling pathway, a hallmark of aging and cancer [43]; cluster 3, the largest cluster, included 65 genes and was enriched in various metabolic pathways, including NAMPT, which supplements eNAMPT-containing exosomes isolated from young mice and significantly extends the lifespan of aged mice [44]; and cluster 5, enriched in RNA transport, included 14 genes, with 12 encoding eukaryotic translation initiation factors (*EIF1*, *EIF2B5*, *EIF2S1*, *EIF2S3*, *EIF3A*, *EIF3D*, *EIF3E*, *EIF3F*, *EIF3G*, *EIF3H*, *EIF3J*, and *EIF5B*). These results indicate that SDEs regulate aging primarily through metabolic pathways.

### DE mRNA and protein correlations in the blood

Exosome proteins were identified as the primary component of total protein in the blood (Fig. 3a). Ten genes (*AMPD2*, *DEK*, *DSP*, *EEF1D*, *HP*, *MMP8*, *NOSTRIN*, *PIBF1*, *PITPNB*, and *TUBA1A*) showed significant differences at both mRNA and SDE protein levels (Additional file 3). The genes *DEK*, *MMP8*, *NOSTRIN*, *PIBF1*, *PITPNB*, and *TUBA1A* also differed at the mRNA and protein levels (Additional file 3), suggesting that these levels were not well-correlated [45]. Four genes (*AMPD2*, *DSP*, *EEF1D*, and *HP*) showed similar trends in mRNA and protein levels during aging (Additional file 3). Notably, *HP* was significantly upregulated in 20–34 years compared with 5–10 years (mRNA: 449-fold change, q-value = 4.25e-04; protein: 6-fold change), aligning with previous studies reporting that HP protein expression is increased in the plasma of aged humans and rats [46]. The antioxidant role of HP protein, preventing hemoglobin-driven oxidative damage [47], provides further support for HP’s important role in aging.

### Biomarkers of aging at the transcriptional level and verification in the brain

**Fig. 5:**
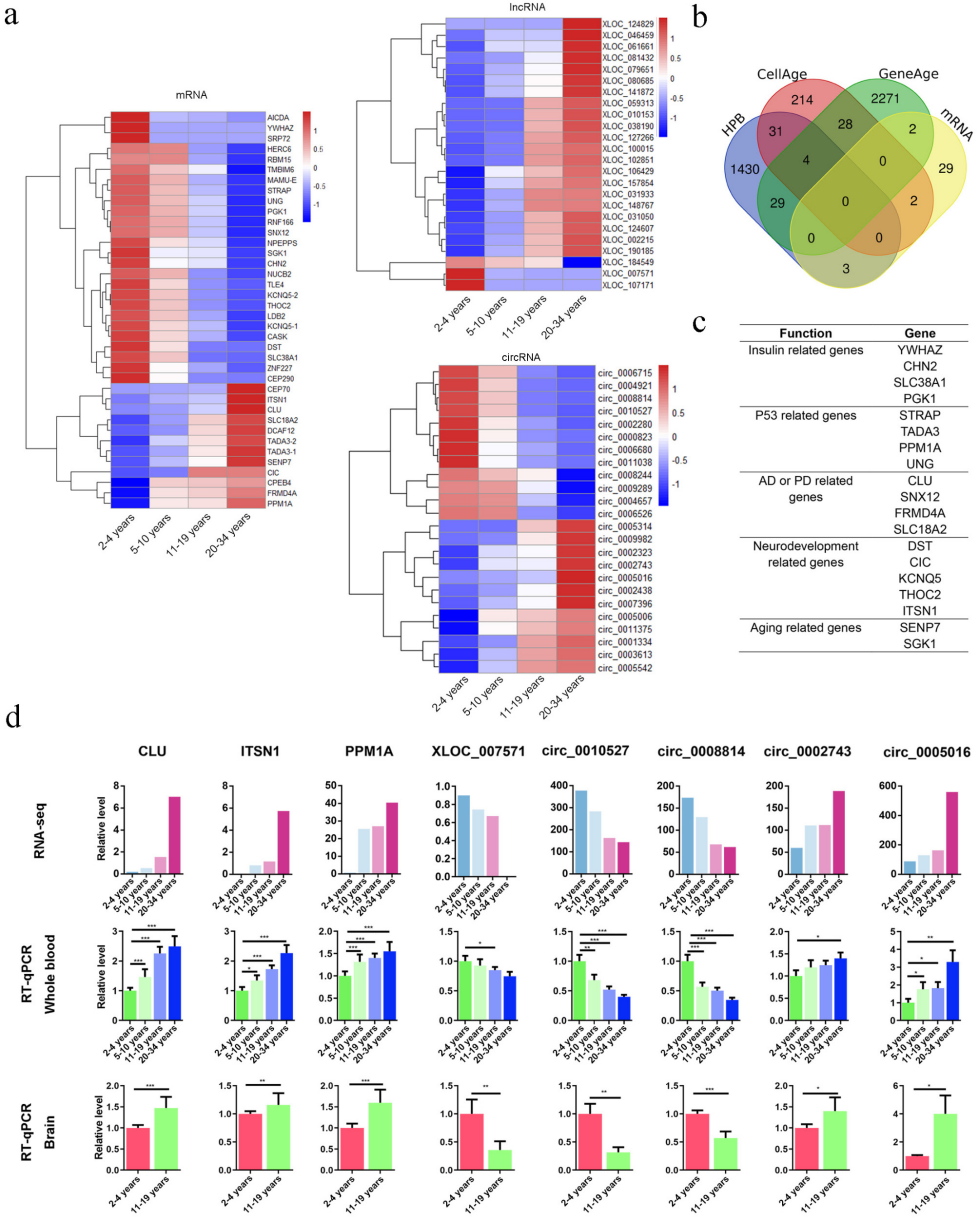
Integrative analyses of the rhesus monkey transcriptome with aging. **a**. DE mRNAs, lncRNAs, and circRNAs according to chronological age. **b**. Venn diagram of aging-associated genes among 36 novel DE mRNAs and those found in CellAge, GenAge, and HPB. **c**. Age-associated genes among 36 novel DE mRNAs. **d**. Barplots of *CLU*, *ITSN1*, *PPM1A*, *XLOC_007571*, *circ_0002743*, *circ_0005016*, *circ_0010527*, and *circ_0008814* expression levels in blood and brain samples across four age groups. *p < 0.05, **p < 0.01, and ***p < 0.001 (two-way ANOVA).

To identify aging biomarkers from mRNAs, lncRNAs, and circRNAs, age-associated upregulated and downregulated genes were filtered from the DE RNAs. In total, 36 DE mRNAs (upregulated: 11; downregulated: 25), 24 DE lncRNAs (upregulated: 21; downregulated: 3), and 24 DE circRNAs (upregulated: 12; downregulated: 12) (Fig. 5a) were detected. Intersection analysis performed between the age-related candidate genes identified in our study and those from previous human, animal, and cell studies (Fig. 5b) revealed seven overlapping genes: *TADA3*, *SLC38A1*, and *NUCB2* in HPB (the transcriptional landscape of age in human peripheral blood [48]); *CLU* and *YWHAZ* in GenAge (the aging gene database); and *SENP7* and *SGK1* in CellAge (the cell senescence gene database). *TADA3* stimulates p53 acetylation and cell senescence induction [49, 50]; *SLC38A1* mediates insulin-regulated glucose metabolism [51]; *NUCB2* plays a crucial role in the hypothalamic pathways regulating food intake and energy homeostasis [52]; *CLU* serum levels increase with age in humans, and *CLU* overexpression extends *Drosophila melanogaster* lifespan [53, 54]; *YWHAZ* regulates insulin sensitivity [55]; *SENP7* is required to promote a permissive chromatin environment for DNA repair [56] and the repression of senp7-induced cell senescence [57]; and *SGK1* overexpression can delay endothelial senescence through the activation of telomerase and reduction of reactive oxygen species levels [58]. Additionally, 13 of the 36 significant age-associated DE mRNAs are involved in pathways related to p53 (*STRAP* [59], *PPM1A* [60], and *UNG* [61]), insulin (*CHN2* [61] and *PGK1* [62]), Alzheimer’s disease or Parkinson’s disease (*SNX12* [63], *FRMD4A* [64, 65], and *SLC18A2* [66]), and neurodevelopment (*DST* [67], *CIC* [68], *KCNQ5* [69], *THOC2* [69], and *ITSN1* [70]) (Fig. 5c).

The expression levels of 16 mRNAs, 4 lncRNAs, and 12 circRNAs, abundant in the blood, were assessed for their potential roles in aging. Both quantitative reverse-transcription PCR (RT-qPCR) and RNA-seq analyses confirmed age-related gene upregulation or downregulation in macaque blood across the four age groups (Additional file 4 and 5). We also investigated these gene expression changes in 2–4 years and 10–19 years macaque brains (Additional file 6 and 7). Interestingly, eight genes (*CLU*, *ITSN1*, *PPM1A*, *XLOC_007571*, *circ_0002743*, *circ_0005016*, *circ_0010527*, and *circ_0008814*) exhibited consistent changes in both blood and brain analyses (Fig. 5d).

### Identification and verification of aging biomarkers at the protein level

**Fig. 6:**
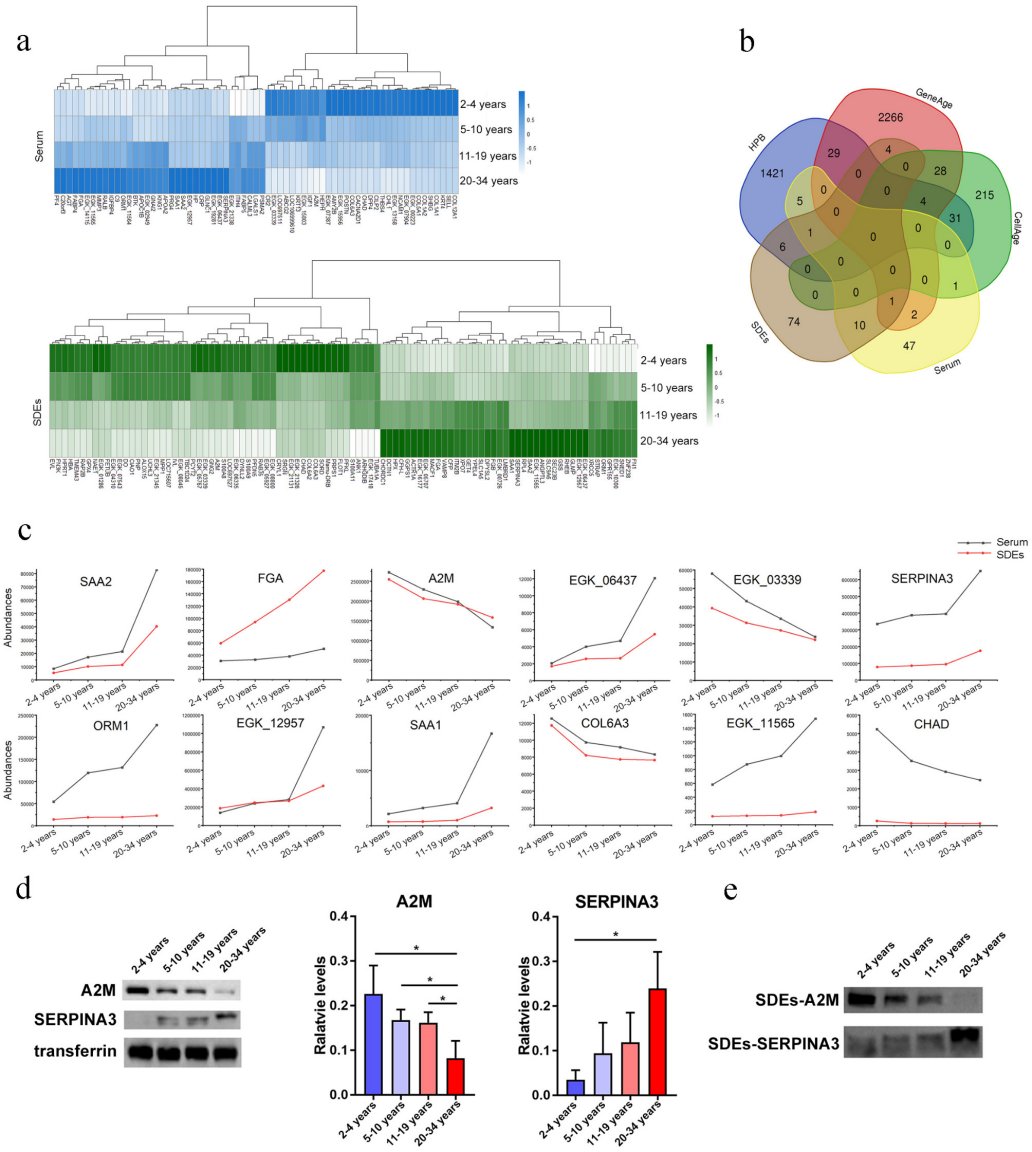
Integrative analyses of serum and SDE proteomes over time in rhesus monkeys. **a**. DE serum and SDE proteins according to chronological age. **b**. Venn diagram of aging-associated genes in serum and SDEs (novel) and in CellAge, GenAge, and HPB. **c**. Twelve DEPs shared among serum and SDEs exhibited the same expression trends. **d**. Western blot analysis of A2M and SERPINA3 in serum, with transferrin used as the loading control. **e**. Expression of A2M and SERPINA3 in SDEs was assessed via western blot analysis

To identify aging biomarkers from blood proteins, age-associated upregulated and downregulated proteins were filtered by their expression levels in serum and SDEs. In serum, 35 and 32 upregulated and downregulated DEPs, respectively, were associated with aging, whereas in SDEs, 42 and 54 upregulated and downregulated DEPs, respectively, were linked to chronological age (Fig. 6a). Intersection analysis between the age-related candidate proteins in serum and SDEs identified in our study and the age-related candidate genes identified in previous human, animal, and cell studies (Fig. 6b) revealed that 10 candidate serum proteins have already been reported: ORM1, NCAM1, HP, DPP4, LGALS1, and CR2 in HPB; CHL1, IGF1, and A2M in GenAge; and AGT in CellAge. The absence of CHL1 shortens yeast cell lifespan [71]; reduced IGF1 expression extends lifespan in rats, and low IGF-1 levels predict life expectancy in exceptionally long-lived individuals [72, 73]; A2M serves as an aging biomarker of human fibroblasts [72, 73]; AGT significantly induces the premature senescence of human vascular smooth muscle cells via the p53/p21-dependent pathway [74]; and NCAM1 is one of the most well-described T-cell aging markers [75, 76]. Additionally, 47 novel candidate aging biomarker DEPs were identified through serum proteomic analysis.

Similarly, in SDEs, 12 candidate proteins were previously reported: ORM1, CHORDC1, RPL4, S100A8, GNG2, EVL, and DPYSL2 in HPB; and XRCC5, GPX4, FN1, GSS, and A2M in GenAge. Mice with XRCC5 deletion exhibit premature aging and a shortened lifespan owing to the protein’s induction of a p53-mediated DNA damage response [75, 76]; RPL4 affects p53 stabilization and activation [77]; GPX4 is an antioxidant defense enzyme that plays a vital role in mitigating the effects of oxidative damage on membrane lipids, and reduced GPX4 levels increase lifespan [77]; GSS is a glutathione synthetase involved in redox regulation and oxidative defense [78]; a lack of FN1 shortens life expectancy [78]; and S100A8 induces autophagy and apoptosis [79]. Additionally, 74 novel candidate aging biomarker DEPs were identified through SDE proteomic analysis. Furthermore, 12 DEPs were shared between serum and SDEs, exhibiting consistent expression trends, possibly owing to their exosome origin (Fig. 6c).

To validate the candidate aging biomarker proteins, proteins identified in serum and SDEs were subjected to western blot analysis. Based on the bioinformatics analysis and the antibodies available in our laboratory, we selected the A2M and SERPINA3 proteins, both DE in serum and SDEs. Validation was conducted using the pooled samples used in quantitative proteomic analyses. In serum and SDEs, A2M exhibited downregulation with chronological age, whereas SERPINA3 showed upregulation during aging (Fig. 6d, e, additional file 8). Western blot data aligned with the proteomics findings.

## Discussion

The average age of rhesus monkeys is approximately 20 years old, at the same time, we referred to the grouping situation of the age of rhesus monkeys in the article published by Liu et al. [26] and two reports about rhesus monkeys [80, 81]. Published literature combined with our experience in raising rhesus monkeys, we consider that before the age of 4 is the development stage of young monkeys, from the age of 5 to 10 they begin to have fertility and reach the peak of reproduction, from the age of 11 the reproductive ability starts to decline, and at the age of 19 most monkeys begin to die naturally. The oldest monkey that we can collect is 34 years old. Therefore, we divide them into 4 groups.

Advances in our understanding of the molecular mechanisms of aging have emphasized the complex nature of this process, although many mechanisms remain unclear [82]. Examining age-related molecular changes in blood offers insights into aging biology [18]. Our analysis of monkey peripheral blood, encompassing mRNAs, lncRNAs, circRNAs, serum proteins, and SDE proteins, revealed novel molecular mechanisms and aging-related biomarkers. The resulting RNA and protein atlas stands as a valuable public resource for researchers.

Certain lncRNAs involved in the induction and maintenance of human aging exhibit highly specific spatial and temporal expression patterns [26, 83, 84]. Comparing gene expression profiles in blood samples, we found that, unlike mRNAs and circRNAs, highly expressed lncRNAs were abundant in 11–19 years. KEGG pathway analysis indicated the association of these lncRNA coexpression genes with several cancer pathways (Fig. 2b), suggesting their more substantial role during aging.

Deep mining of aging transcriptomes revealed undulating changes during aging in monkeys, consistent with findings in humans [18]. Although linear patterns have received extensive attention, nonlinear trajectories are less well-studied [18]. However, our KEGG analysis showed both linear and nonlinear patterns enriched in aging-related biological pathways. For example, among mRNAs, the mTOR signaling pathway was enriched in linear patterns, whereas the insulin signaling pathway and FoxO signaling pathway were enriched in nonlinear patterns (Fig. 2d). The inclusion of both types of changes enhances the analysis of aging in longitudinal datasets.

Exosomes are widespread throughout the body and implicated in aging [85, 86]. We found a considerable overlap of proteins between the serum proteome and SDE proteome, with these overlapping proteins derived primarily from exosomes, indicating that exosome proteins are the main components of serum proteins. Integrating serum and SDE proteomes, we established a relatively comprehensive rhesus monkey blood proteome database. We discovered 1270 and 2160 proteins in serum and SDEs respectively. Even though the number of proteins in serum is lower than that in SDEs, which appears counterintuitive as serum contains SDEs. However, since the content of SDEs in serum is extremely low, it is not necessarily possible to detect SDEs proteins. The SDEs proteins used for detection are obtained via separation and concentration, so it is comprehensible that the number of proteins in SDEs is higher than that in serum. Additonally, in the work of Yang et al. [87] , the researchers compared the protein changes in serum and serum exosomes. 271 and 430 proteins were screened and identified in serum and serum exosomes respectively, and further differential analysis indicated that the number of differential proteins in serum exosomes was more than that in serum. This is similar to the results of our proteomics analysis of serum and SDEs, although there are differences in the number of proteins, which might be caused by factors such as sample source, experimental methods and techniques. Given the limitations of current proteomic techniques in detecting trace proteins in blood samples, and considering that SDE proteins play key roles in the blood [88], SDEs may be more suitable than serum for blood-based proteome studies.

Assessing the aging process and intervention efficacy requires biomarkers, with the blood serving as a sensitive indicator of functional aging [89, 90]. This study aimed to identify aging biomarkers in blood samples, considering a broad range of circRNAs, lncRNAs, and SDE proteins, unlike previous mRNA-focused or plasma protein-centric approaches [25, 48, 91]. Ultimately, 84 RNAs and 163 proteins emerged as candidate aging biomarkers. Of these, 27 genes have been associated with aging in HPB, GenAge, and CellAge. The remaining genes and proteins align with functions associated with known aging mechanisms, including metabolic function, the p53 pathway, DNA repair, insulin signaling, and the mTOR pathway [92]. Notably, changes in eight genes (*CLU*, *ITSN1*, *PPM1A*, *circ_0002743*, *circ_0005016*, *XLOC_007571*, *circ_0010527*, and *circ_0008814*) were consistent between blood and brain analysis (Fig. 5d) according to RT-qPCR results. The work of Trougakos et al. [53] and Baralla et al. [93] have demenstrated an increase of CLU protein in plasma sample during aging occurs until 99 years in population, but decreased in centenarians. This may be related to the CLU function as a sensitive biosensor of oxidative stress [94]. CLU involvement in reverse cholesterol transport and it shows a positive correlation with total cholesterol and low-density lipoprotein, which may be due to the increasing cardiovascular risk with age. *ITSN1* plays an important role in brain development. Knockout mice show defects in neuronal migration and synaptic plasticity in the hippocampus and cortex, as well as abnormal secretion and transportation of synaptic vesicles [95], and changes also occur in spatial learning and memory [96]. In the population, ITSN1 is associated with the progression of several neurodegenerative disorders. PPM1A dephosphorylates and inactivates the AMPK pathway [97]. The characteristic of degenerative brain disease is the abnormal activation of AMPK, which can affect the synaptic function related to AD [98], and the progression of amyotrophic lateral sclerosis (ALS) [99] and Huntington’s disease (HD) [100]. Although the relationship between *ITSN1* and *PPM1A* and aging has not yet been explored, diseases related to it, such as Alzheimer’s disease, usually occur along with aging, which may indicate that *ITSN1* and *PPM1A* has a certain correlation with aging. RNA or protein changes in the blood reflect various aspects of aging in different cell types and tissues [18]. Through retrieval, we found the *CLU* gene in the database related to human or cellular aging. The *CLU* gene has a close relationship with aging, and the plasma CLU protein level in the population shows a positive correlation with age. This result support the screening of the *CLU* gene as an aging marker, indicating that the aging marker screening process we established is reliable. Meanwhile, we have discovered several genes that are not in human aging databases, including *ITSN1* and *PPM1A*, one lncRNA, and four circular RNAs, suggesting that they may become new potential aging markers. Therefore, these eight genes may serve as blood biomarkers for detecting brain aging. However, larger-scale blood- and brain-based studies, especially in humans, are necessary to validate these genes.

## Conclusions

This study provides novel insights into the molecular mechanisms underlying aging in healthy blood through comprehensive transcriptome and proteome analyses. Additionally, eight genes serving as potential blood biomarkers for detecting brain aging are identified. Overall, this research enhances our understanding of the molecular basis of aging and offers new aging biomarkers.

### Electronic supplementary material

Below is the link to the electronic supplementary material.

Supplementary Material 1

Supplementary Material 2

Supplementary Material 3

Supplementary Material 4

Supplementary Material 5

Supplementary Material 6

Supplementary Material 7

Supplementary Material 8

Supplementary Material 9

Supplementary Material 10

Supplementary Material 11

Supplementary Material 12

Supplementary Material 13

Supplementary Material 14

Supplementary Material 15

Supplementary Material 16

Supplementary Material 17

Supplementary Material 18

Supplementary Material 19

Supplementary Material 20

## Data Availability

RNA-seq raw sequencing data are available in SRA (BioProject No. PRJNA791697: https://www.ncbi.nlm.nih.gov/bioproject/PRJNA791697). MS proteomics data was deposited into ProteomeXchange under accession numbers PXD030287 (https://proteomecentral.proteomexchange.org/cgi/GetDataset?ID=PXD030287) and PXD030289 (https://proteomecentral.proteomexchange.org/cgi/GetDataset?ID=PXD030289).

## Abbreviations

BCA: Bicinchoninic acid
DE: Differentially expressed
DEP: Differentially expressed proteins
FDR: False discovery rate
GO: Gene Ontology
HAP: High-abundance proteins
KEGG: Kyoto Encyclopedia of Genes and Genomes
MS: Mass spectrometry
PBS: Phosphate-buffered saline
PCR: Polymerase chain reaction
SDE: Serum-derived exosomes
STEM: Short Time-series Expression Miner
TBS: Tris-buffered saline
TMT: Tandem mass tag
TPM: Transcripts per million
