# Supplementary figures and images for: Analysis of the aging-related biomarker in a nonhuman primate model using multilayer omics
Source: BMC Genomics. 2024 Jun 26;25:639. doi: 10.1186/s12864-024-10556-z (PMC11209966; doi:10.1186/s12864-024-10556-z)

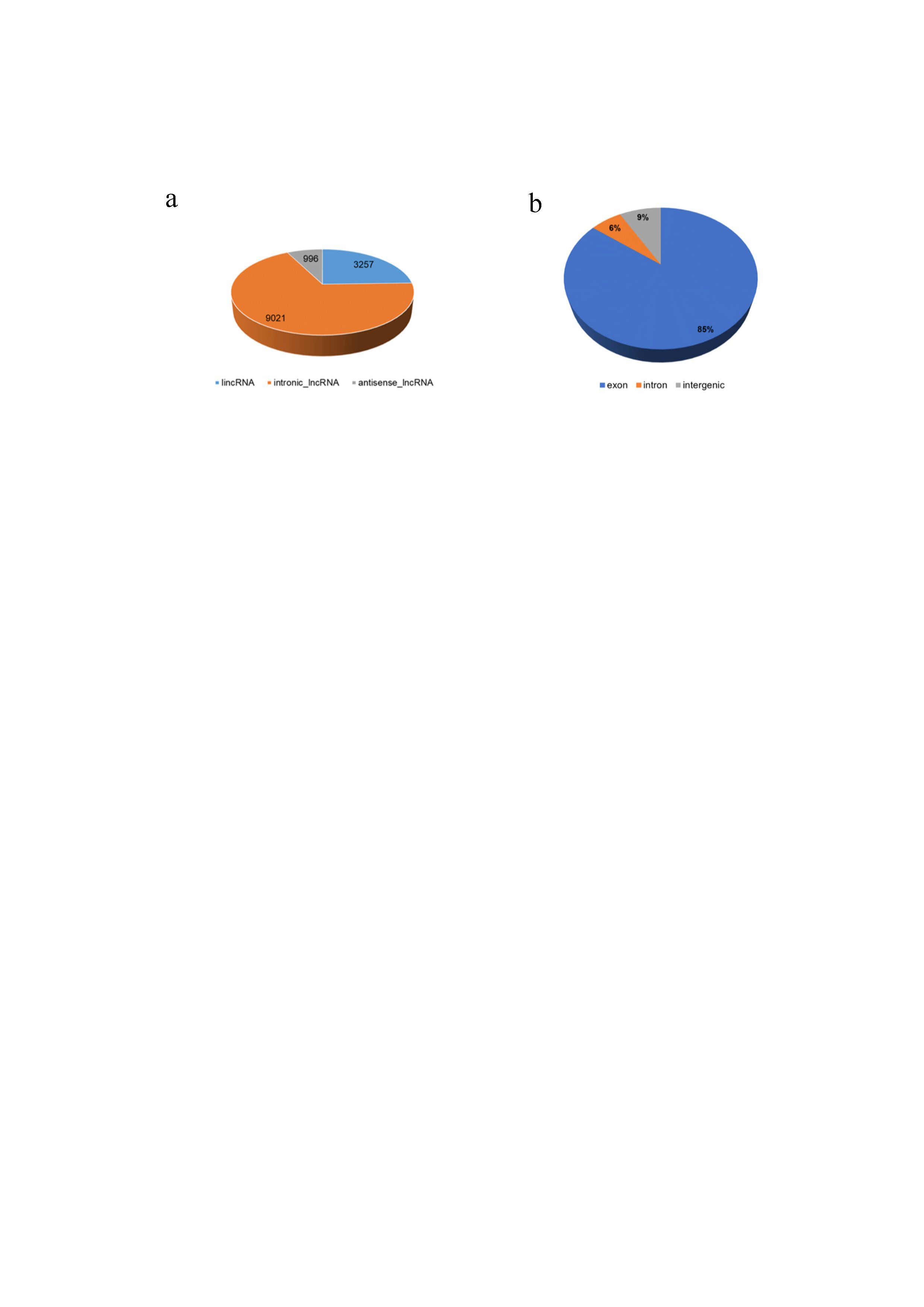

Supplement: Supplementary file 1 — Supplementary Material 1 [file 12864_2024_10556_MOESM1_ESM.jpg]

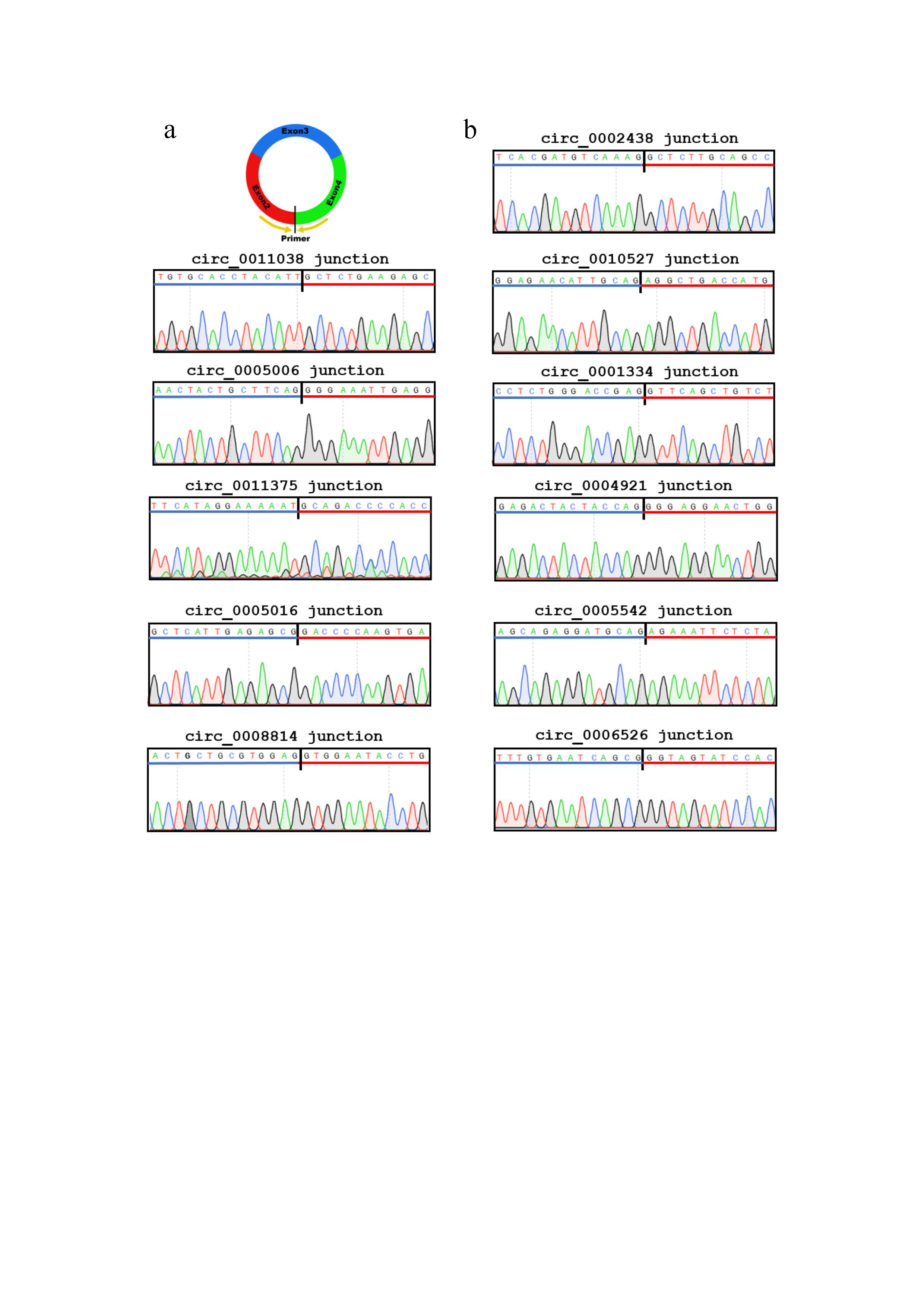

Supplement: Supplementary file 2 — Supplementary Material 2 [file 12864_2024_10556_MOESM2_ESM.jpg]

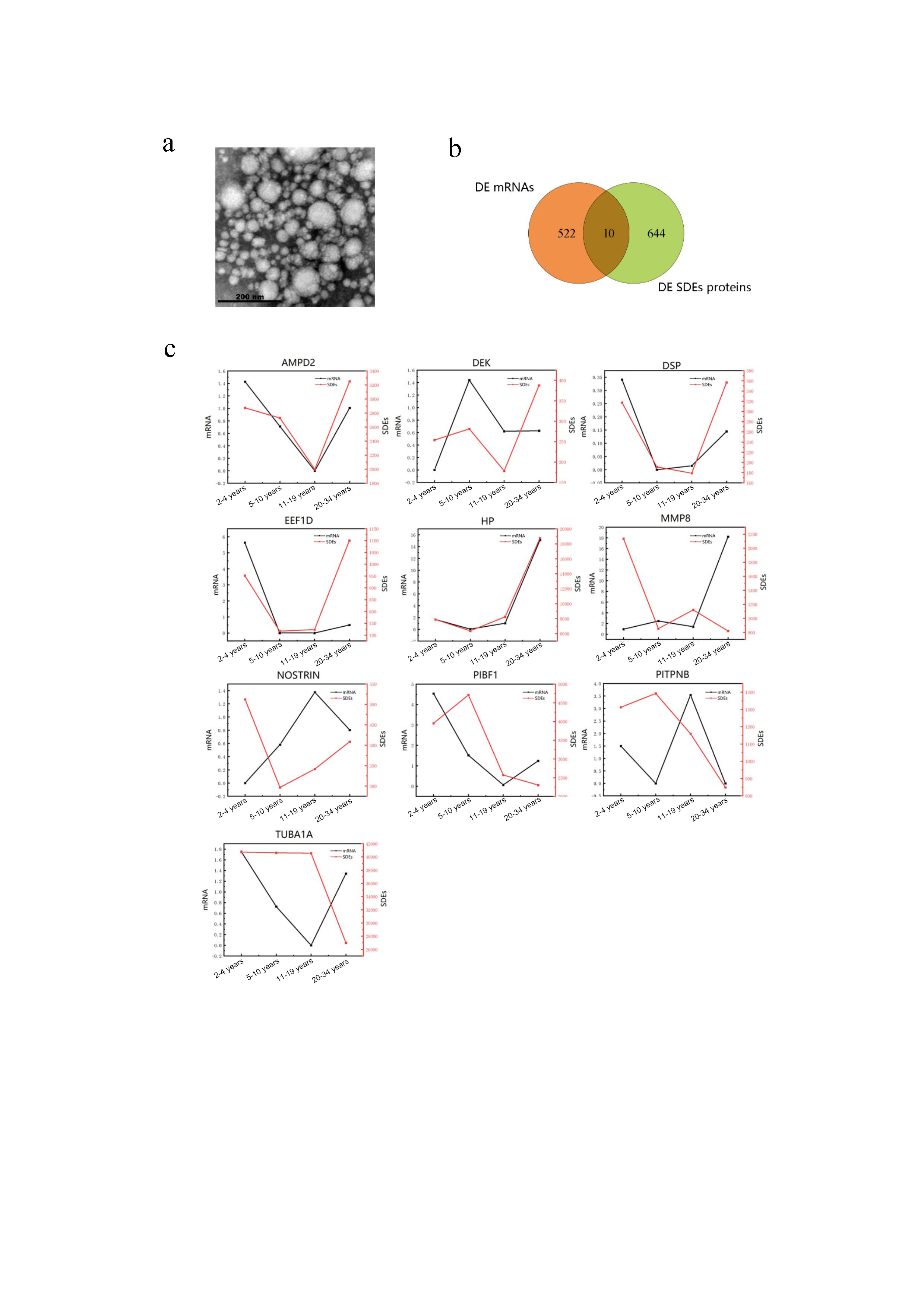

Supplement: Supplementary file 3 — Supplementary Material 3 [file 12864_2024_10556_MOESM3_ESM.jpg]

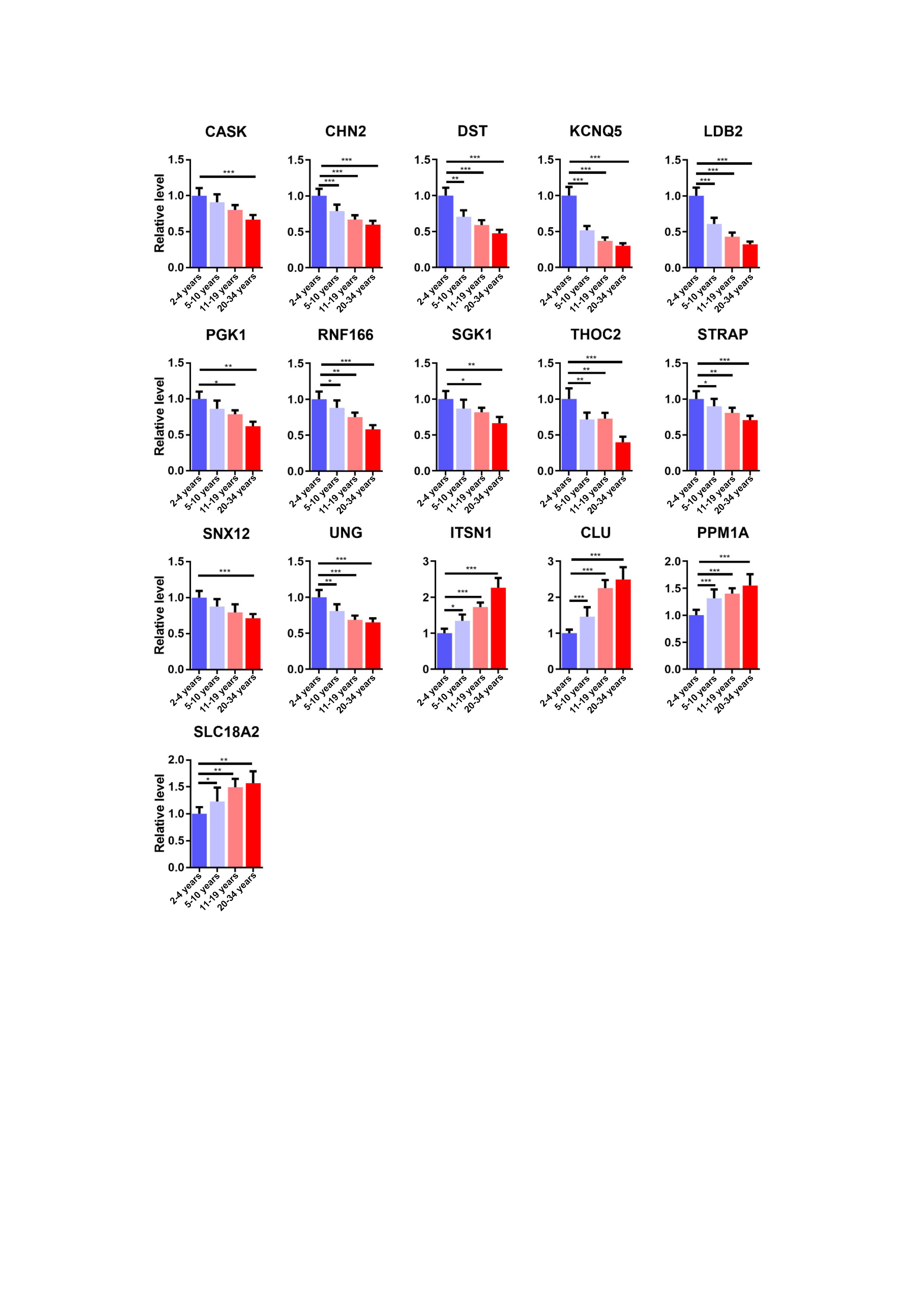

Supplement: Supplementary file 4 — Supplementary Material 4 [file 12864_2024_10556_MOESM4_ESM.jpg]

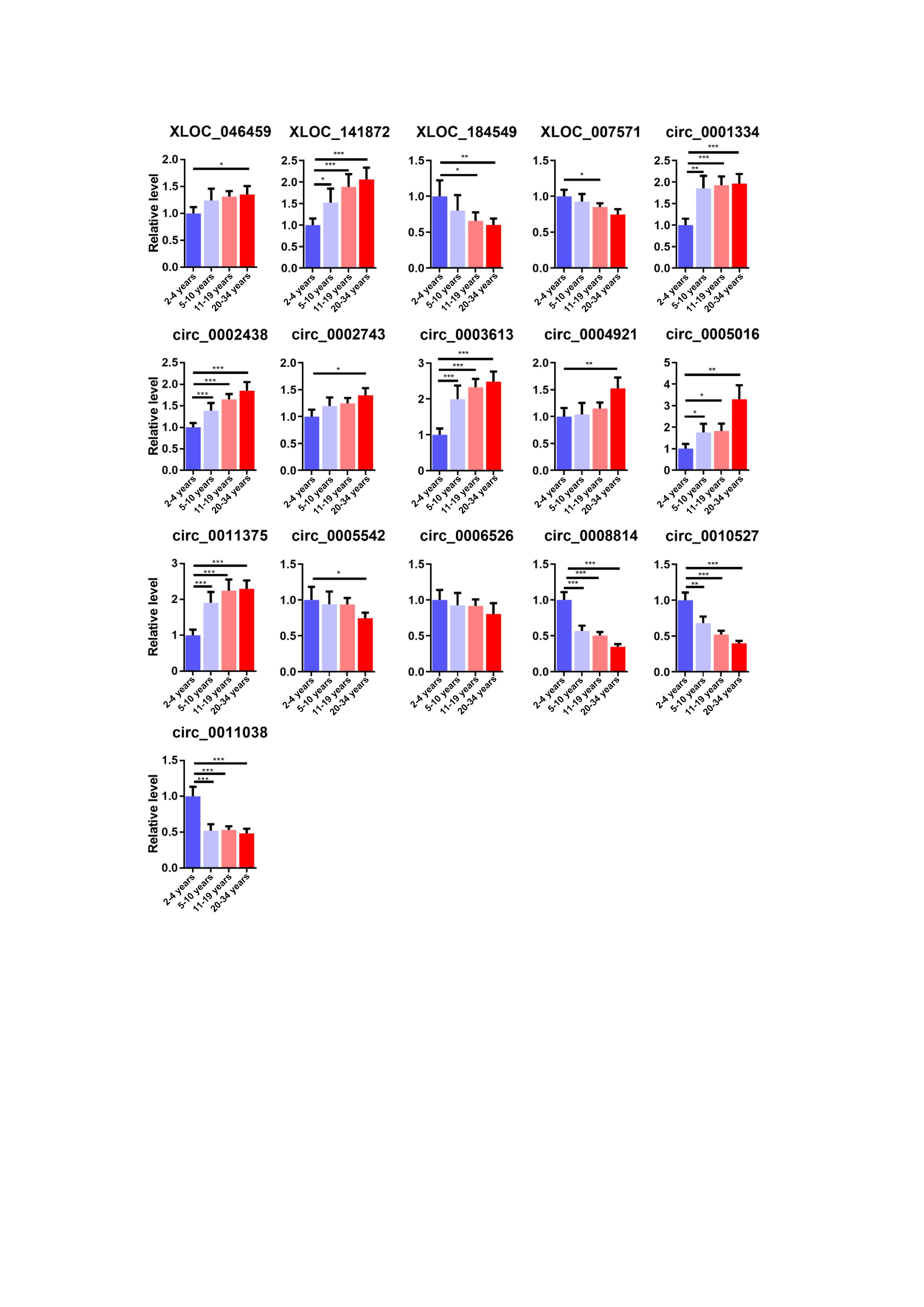

Supplement: Supplementary file 5 — Supplementary Material 5 [file 12864_2024_10556_MOESM5_ESM.jpg]

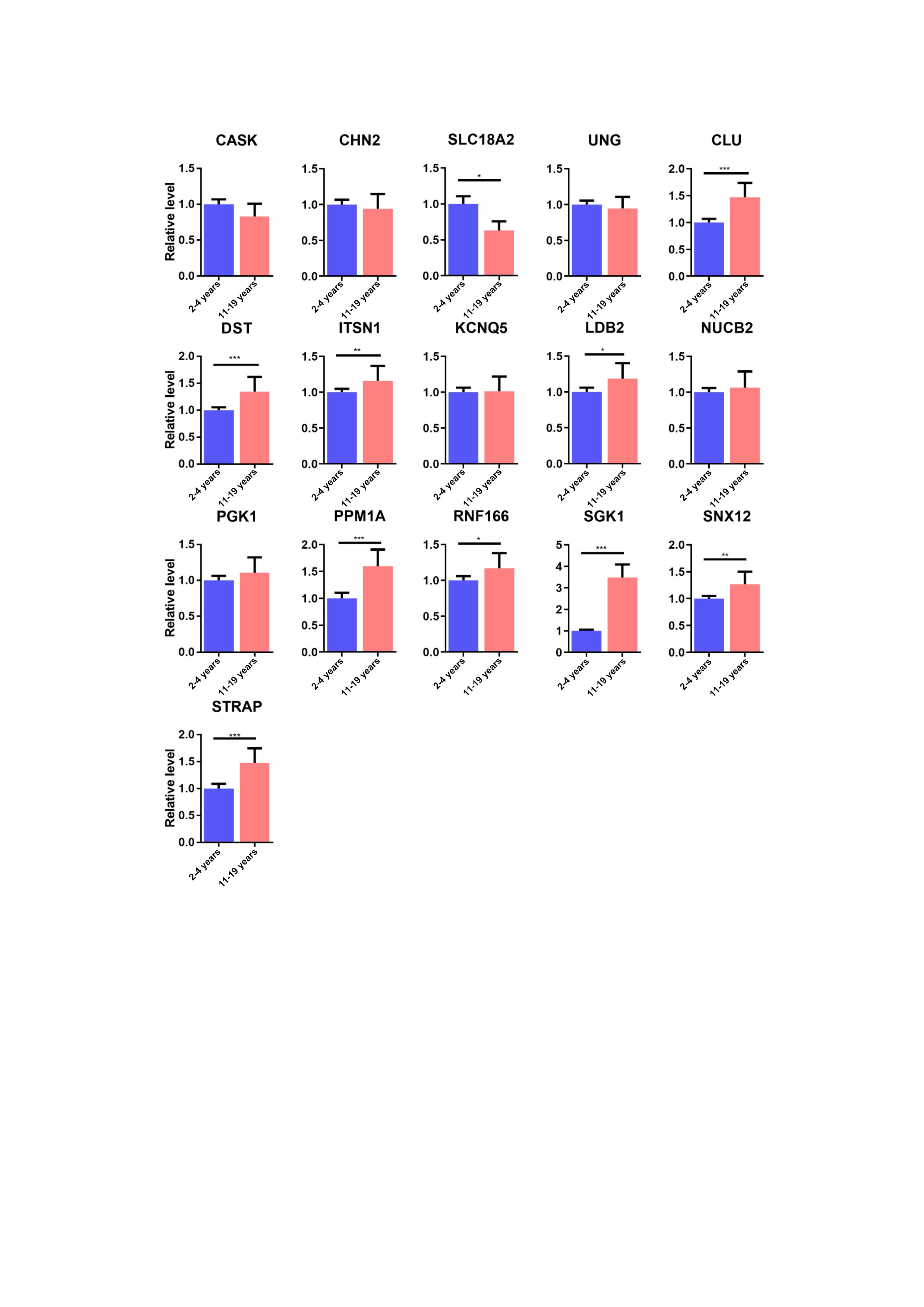

Supplement: Supplementary file 6 — Supplementary Material 6 [file 12864_2024_10556_MOESM6_ESM.jpg]

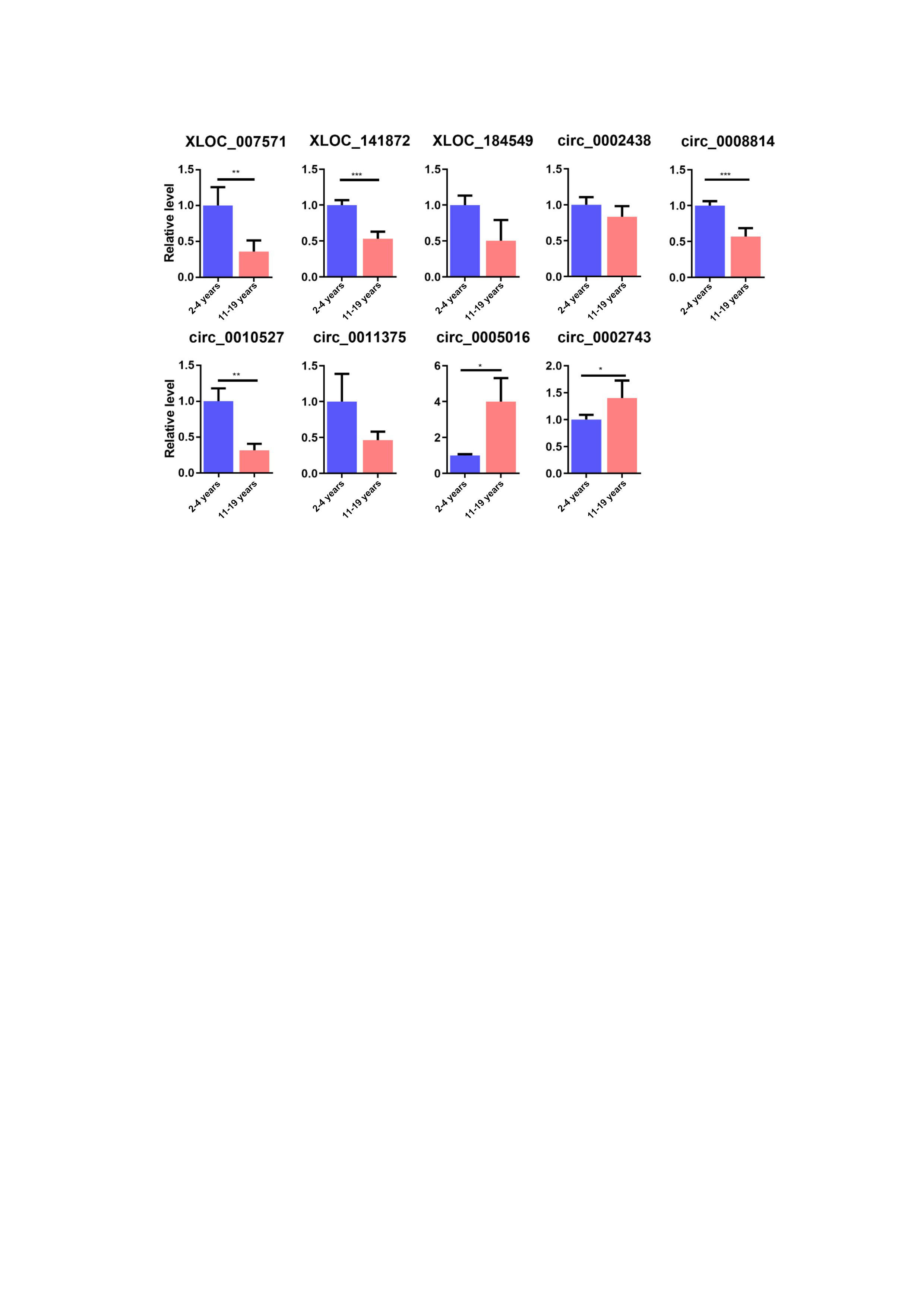

Supplement: Supplementary file 7 — Supplementary Material 7 [file 12864_2024_10556_MOESM7_ESM.jpg]

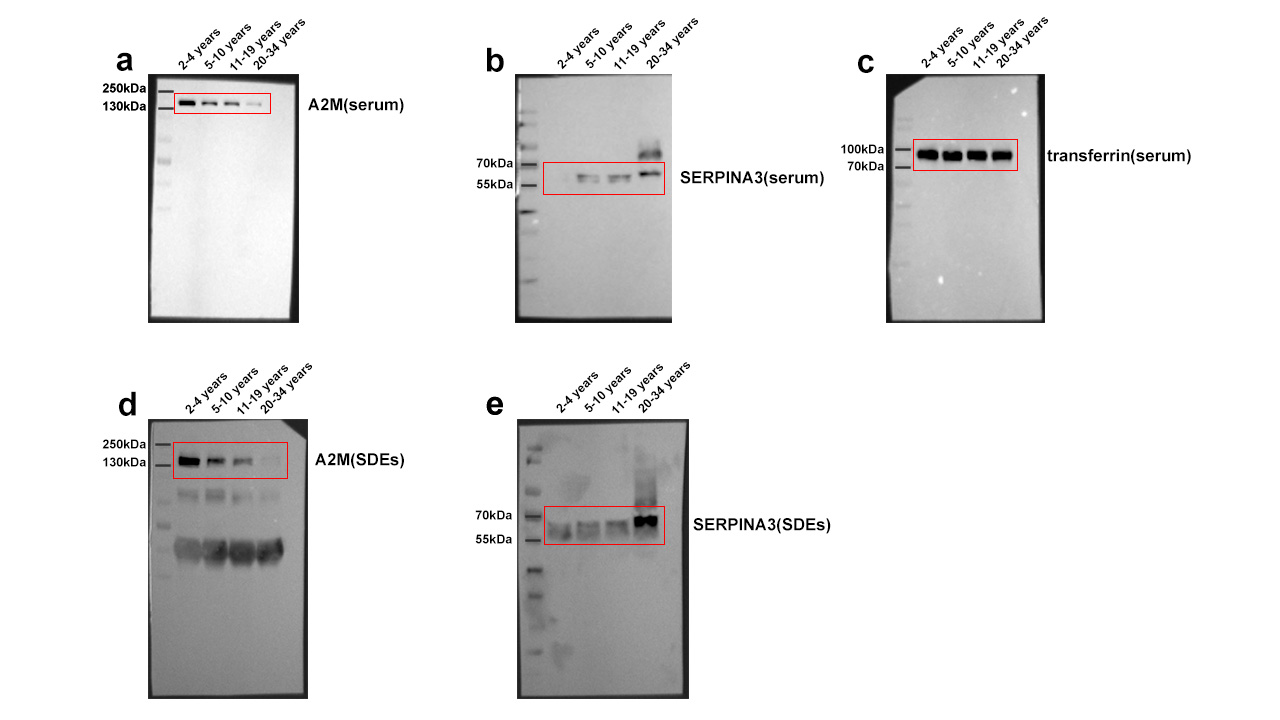

Supplement: Supplementary file 8 — Supplementary Material 8 [file 12864_2024_10556_MOESM8_ESM.jpg]
