# Supplementary material for: Analysis of the aging-related biomarker in a nonhuman primate model using multilayer omics
Source: BMC Genomics. 2024 Jun 26;25:639. doi: 10.1186/s12864-024-10556-z (PMC11209966; doi:10.1186/s12864-024-10556-z)
Supplement: Supplementary file 20 — Supplementary Material 20 [file 12864_2024_10556_MOESM20_ESM.docx]

**Supplementary Information**

**Additional file 1: Fig. S1.**

Information regarding lncRNAs and circRNAs (a) Proportion of different lncRNA types among all lncRNAs. (b) Proportion of different circRNA types among all predicted circRNAs.

**Additional file 2: Fig. S2.**

Validation of circRNA-sequencing results. (a) Primer patterns of circRNAs. Blocks represent exons, green arrows represent primers, and the black vertical line represents the circRNA back‐spliced junction. (b) Back‐spliced junctions of 11 randomly selected circRNAs were confirmed using Sanger sequencing. Black vertical line represents back‐spliced junction sites, and the blue and red horizontal lines indicate the 5′ and 3′ ends of the circRNA sequence, respectively.

**Additional file 3: Fig. S3.**

Information regarding SDEs and their correlation with mRNAs (a) Electron microscope image of SDEs. (b) Overlap of DE mRNAs and DE SDEs proteins. (c) mRNA and protein levels for 10 genes.

**Additional file 4: Fig. S4.**

RT‐qPCR validation of DE mRNAs in blood samples. RT‐qPCR results validated the relative expression levels of 16 randomly selected DE mRNAs at the transcriptional level in a separate cohort of blood samples distinct from the transcriptome cohort (n = 9).

**Additional file 5: Fig. S5.**

RT‐qPCR validation of DE lncRNAs and circRNAs in blood samples. RT‐qPCR results validated the relative expression levels of 4 randomly selected DE lncRNAs and 12 randomly selected DE circRNAs at the transcriptional level in a separate cohort of blood samples from the transcriptome cohort (lncRNA n = 9; circRNA n = 5).

**Additional file 6: Fig. S6.**

RT‐qPCR validation of DE mRNAs in brain samples. RT‐qPCR results validated the relative expression levels of 16 DE mRNAs at the transcriptional level in brain samples (n = 6).

**Additional file 7: Fig. S7.**

RT‐qPCR validation of DE lncRNAs and circRNAs in blood samples. RT‐qPCR results validated the relative expression levels of 4 DE lncRNAs and 12 DE circRNAs at the transcriptional level in blood samples (n = 6).

**Additional file 8: Fig. S8.**

Full uncropped Blots images. **a-c.** The expression of A2M, SERPINA3 and transferrin in serum. **d-e.** The expression of A2M and SERPINA3 in SDEs.

**Additional file 9: Table S1.**

Blood biochemical indexes of rhesus monkey transcriptome samples.

**Additional file 10: Table S2.**

Sequencing and alignment information for all RNA-seq samples.

**Additional file 11: Table S3.**

Expression level (FPKM) for each mRNA gene detected in our study.

**Additional file 12: Table S4.**

Expression level (FPKM) for each lncRNA gene detected in our study.

**Additional file 13: Table S5.**

Expression level (TPM) for each circRNA gene detected in our study.

**Additional file 14: Table S6.**

Differentially expressed genes and six groups defined via STEM in the samples.

**Additional file 15: Table S7.**

KEGG enrichment of six groups via KOBAS (p < 0.05).

**Additional file 16: Table S8.**

Serum proteomics.

**Additional file 17: Table S9.**

Serum exosome proteomics.

**Additional file 18: Table S10.**

Primers used for DE RNA validation.

**Additional file 19: Table S11.**

Primers used for circRNA sequence validation.
